# Spiked gold nanotriangles: formation, characterization and applications in surface-enhanced Raman spectroscopy and plasmon-enhanced catalysis†

**DOI:** 10.1039/d0ra00729c

**Published:** 2020-02-25

**Authors:** Ferenc Liebig, Radwan M. Sarhan, Matias Bargheer, Clemens N. Z. Schmitt, Armen H. Poghosyan, Aram A. Shahinyan, Joachim Koetz

**Affiliations:** Institute for Chemistry, University of Potsdam Karl-Liebknecht-Strasse 24-25, Haus 25 14476 Potsdam Germany koetz@uni-potsdam.de +49 331 977 5220; Chemistry Department, Faculty of Science, Cairo University Cairo 12613 Egypt; Institute for Physics, University of Potsdam Karl-Liebknecht-Strasse 24-25, Haus 27 14476 Potsdam Germany; School of Analytical Sciences Adlershof (SALSA), Humboldt-Universität zu Berlin Albert-Einstein-Str. 5-9 10099 Berlin Germany; Department of Biomaterials, Max Planck Institute of Colloids and Interfaces Am Mühlenberg 1 14476 Potsdam Germany; International Scientific-Educational Center of National Academy of Sciences M. Baghramyan Ave. 24d 0019 Yerevan Armenia

## Abstract

We show the formation of metallic spikes on the surface of gold nanotriangles (AuNTs) by using the same reduction process which has been used for the synthesis of gold nanostars. We confirm that silver nitrate operates as a shape-directing agent in combination with ascorbic acid as the reducing agent and investigate the mechanism by dissecting the contribution of each component, *i.e.*, anionic surfactant dioctyl sodium sulfosuccinate (AOT), ascorbic acid (AA), and AgNO_3_. Molecular dynamics (MD) simulations show that AA attaches to the AOT bilayer of nanotriangles, and covers the surface of gold clusters, which is of special relevance for the spike formation process at the AuNT surface. The surface modification goes hand in hand with a change of the optical properties. The increased thickness of the triangles and a sizeable fraction of silver atoms covering the spikes lead to a blue-shift of the intense near infrared absorption of the AuNTs. The sponge-like spiky surface increases both the surface enhanced Raman scattering (SERS) cross section of the particles and the photo-catalytic activity in comparison with the unmodified triangles, which is exemplified by the plasmon-driven dimerization of 4-nitrothiophenol (4-NTP) to 4,4′-dimercaptoazobenzene (DMAB).

## Abstract 2

We show the formation of metallic spikes on the surface of gold nanotriangles (AuNTs) by using the same reduction process which has been used for the synthesis of gold nanostars leading to a higher SERS enhancement by a factor of 75.

## Graphical abstract

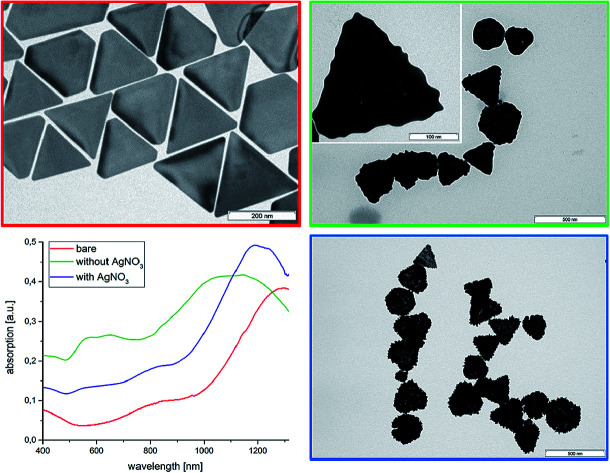

## Introduction

Gold nanoparticles have received a great deal of attention due to their special optical properties.^1,2^ One special feature of noble metals on the nanometer scale is the localized surface plasmon resonance (LSPR).^3,4^ The LSPR strongly depends on the shape and size of the gold nanoparticles, *e.g.*, nanospheres,^5^ nanorods,^6^ nanotriangles,^7,8^ nanostars,^9–12^ or multibranched nanoparticles,^13^ and opens new fields of application.^14,15^ Asymmetric particles allow for creating very large absorption and scattering cross sections in the near-infrared (NIR) region, which is of special interest for surface enhanced Raman scattering (SERS) applications in biological systems. Gold nanostars with their spike tips are of special relevance in this field.^16–19^ Liz-Marzan *et al.* have shown that a Raman active molecule, sandwiched between the spike tip and a plasmonic surface, can enhance the SERS signal up to two orders of magnitude.^19^ Not only the sharpness of the tips is of relevance, but also the arrangement of the spikes to each other which tunes the plasmon resonance. Sheen Mers *et al.* studied the effect of spike length on the SERS signal enhancement^18^ and Atta *et al.*^10^ investigated the seed mediated growth of 6-branched gold nanostars with very thin spikes up to a length of 100 nm. The interplay of surfactant, ascorbic acid (AA), AgNO_3_ and seed concentrations are of high importance for the crystallization of the spikes. Upon excitation with light at the tip apexes of nanostars large electromagnetic fields can be localized, which can be exploited by SERS.^19^

Nanorods and nanotriangles are another class of anisotropic nanoparticles which offer excellent properties in electrocatalysis and Raman scattering due to the large electromagnetic fields at the ends of nanorods^20^ or vertices of nanotriangles.^8,21^ For gold nanotriangles (AuNTs) the sharpness of the edges and tips is of special relevance for improving the enhancement factor, and therefore, the application in catalysis. AuNTs could be produced by using different strategies, *e.g.*, by a silver-free growth of CTAC-coated seeds in the presence of iodide anions^7^ or a seedless synthesis through oxidative etching.^22^ Note that different chemical compounds can be used as SERS substrates, *e.g.*, graphene oxide/AgCo composite nanosheets as efficient catalysts for dimerizing *p*-nitrothiophenol.^23^ However, a novelty of gold-based SERS-active nanostructures is their utilization in biomedical sensing.^24^ AuNTs with an absorption maximum at about 1200 nm, in the second window for *in vivo* imaging,^25^ are of special relevance in this field of research.^26^

Taking this into account, an extraordinary enhancement effect can be expected, when the spike and anisotropic strategies come together. First examples of such a concept were shown by Liz-Marzan *et al.* by growing tips on gold nanorods.^27^ Due to the antenna effect from the spikes higher SERS signals were obtained. Recently, it was shown by different groups that islands produced in the seed-mediated overgrowth^28^ or by a partial surface passivation^29^ of nanotriangles lead to excellent properties in surface-enhanced Raman scattering.

Here we present a new family of spiked gold nanotriangles (SAuNTs) based on ultra-flat (7.5 nm) nanotriangles^8,30^ decorated with gold spikes. The idea is to crystallize gold nanoparticles directly on the {111} gold surface of nanotriangles under similar conditions already used for making nanostars. While the reduction of Au with AA in the AOT (dioctyl sodium sulfosuccinate) shell of the nanotriangles leads to an undulated surface modification, adding the directing agent AgNO_3_ transforms the undulations into sharp spikes growing on the platelet surface. As a result, SAuNTs with a 75 times higher enhancement factor in SERS experiments become available.

## Experimental section

### Materials

Tetrachloroauric(iii)acid (HAuCl_4_·3H_2_O), silver nitrate (AgNO_3_), dioctyl sodium sulfosuccinate (AOT) (98%) and 4-nitrothiophenol (4-NTP) were purchased from Sigma Aldrich. l(+)-Ascorbic acid (AA) (≥99%) were obtained from Roth. Milli-Q Reference A+ water was used in all experiments. The phospholipid PL90G with a purity >97.3% was obtained from Phospholipid GmbH.

### Methods

A Shimadzu UV-2600 spectrophotometer was used in the wavelength range between 200 and 1400 nm to obtain UV-vis-NIR absorption spectra. The morphology of the different gold nanoparticles was identified by using a transmission electron microscope JEM-1011 (JEOL) at an acceleration voltage of 80 kV as well as the JEM-2200 FS (JEOL) at 200 kV for high resolution (HRTEM), fast Fourier transformation (FFT) and element mapping (EDX measurements). For obtaining SEM micrographs a ZEISS Supra 55PV scanning electron microscope at an acceleration voltage of 2 kV was used. The zeta potential was determined with a Malvern Nano Zetasizer 3600. The SERS performance was assessed using a confocal Raman microscope Alpha 300 (WITec) coupled with laser excitation at wavelength of 785 nm. The 2 and 10 mW laser beam was focused on the sample through a Nikon 20× objective lens. The Raman spectra were acquired with a thermoelectrically cooled Andor CCD detector DU401A-BV placed behind the spectrometer UHTS 300 from WITec with a spectral resolution of 3 cm^−1^, and were recorded with an integration time of 2 seconds, which is also the exposure time of the laser beam. The Raman band of the silicon wafer at 520 cm^−1^ was used to calibrate the spectrometer.

### Nanoparticle synthesis

#### Synthesis of AuNTs

The synthesis of the AuNTs was achieved according to our recently published protocol.^8^ A vesicle phase containing 0.5 wt% phospholipid PL90G and 0.5 wt% AOT solution was produced and subsequently mixed with a 2 mM tetrachloroaurate precursor solution. After shaking 10 mL of the vesicular template phase (containing 0.05 g PL90G; 0.05 g AOT; 9.9 g water) for two days, 30 mL of the 2 mM gold chloride solution were added. After heating this mixture 45 minutes at 45 °C, a polydisperse gold nanoparticle solution was obtained. For the separation of the AuNTs from spherical ones, a depletion flocculation was performed by adding a 0.02 M AOT solution. After 3 washing and centrifugation steps (10 minutes at 4000 rpm) the resulting green colored AuNTs solution with a zeta potential of −59 mV was used for further experiments. The AuNTs solution concentration was determined by SAXS and is approximately 0.16 mg mL^−1^ with an estimated uncertainty of 5%.

#### Synthesis of SAuNTs

The AuNTs solution was 4 times diluted to a used stock solution concentration of 0.04 mg mL^−1^. To 300 μL of the AuNTs stock solution we have added 900 μL of a 2 mM gold chloride precursor solution and 45 μL of a 2.5 mM AgNO_3_ solution. For the reduction process 600 μL of a 0.1 M AA solution was added under fast stirring to ensure a homogenous distribution of the reduction agent leading to a monodisperse spike formation. Because of the very fast reaction, the stirring time was 30 seconds. The samples were characterized by UV-vis-NIR spectroscopy, SEM and TEM microscopy.

#### SAuNTs monolayer formation and surface functionalization with Raman active linker molecules

For SERS measurements, 50 μL of the final SAuNTs solution was dropped on a silicon wafer. The concentration of the final SAuNTs solution was not determined, but after the centrifugation steps and the removement of the sphere- or star-like ones, the concentration should be almost the same as the stock AuNTs solution. The addition of 25 μL of the ethanol–toluene-mixture (volume ratio 5 : 1) to the droplet leads to a film casting process as described by us earlier.^31^ The evaporation process was done at room temperature under a covered glass with air slits at the bottom. The silicon wafer was immersed in 10 mM 4-nitrothiophenol (4-NTP) ethanolic solution for 3 hours after the solvent evaporation, and was washed several times in ethanol to remove the physisorbed molecules.

### Construction and MD simulation details

#### MD simulations of the adsorption of AA at the surface of AOT-stabilized AuNTs

We build on the MD simulations the AOT bilayer composed of 84 molecules adsorbed on Au(111) facets.^32,33^ After addition of 50 AA molecules this system was optimized for 5000 steps. A 100 ns production run was carried out and the last 50 ns of production trajectories were used for data collection and further analysis. Note that the statistic independent run (100 ns) was also done.

Two simulations were carried out using GROMACS software package and the CHARMM27 all-atom force field protocols for the AOT surfactant, described by Abel *et al.*^34^ Note that the GOIP-CHARMM all atom force field was applied to describe the gold surface,^35^ already used by us previously.^32,33^ The forcefield for AA molecules was retrieved with the CGenFF database server,^36^ which generates the CHARMM force field for small organic molecules. In two simulations, the SPC model^37^ was used for water molecules. The room temperature was maintained by applying a Nose–Hoover thermostat,^38^ and the temperatures for all components were controlled independently. The LINCS approach^39^ was applied to fix all bonds. The PME method^40^ (truncation at 1.2 nm) was used for electrostatic interactions and the van-der-Waals interactions were cut at 1.2 nm, too. The equation of motion was integrated using the Verlet-leapfrog approach^41^ with a timestep of 1 fs.

The clusters available at IIAP NAS RA^42^ were used. Additional computational resources were obtained from http://bioinformatics.am. All visualizations in the text have been represented by VMD graphical package.^43^

#### MD simulations of the adsorption of AA at the surface of a gold cluster

A gold sphere consisting of 144 Au atoms^44^ was solvated in bulk water in the presence of 50 AA molecules. Further steps and simulation details were the same as described above. The production run for 50 ns was carried out.

## Results and discussion

### Formation of SAuNTs

**Scheme 1:**
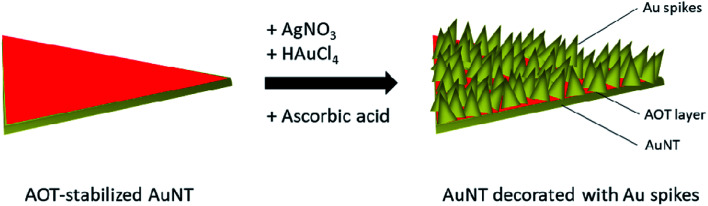
Approach to SAuNTs.

The general concept for the synthesis of SAuNTs is shown in Scheme 1, where AuNTs have been decorated with spikes in presence of AgNO_3_, HAuCl_4_ and AA.

### AuNTs synthesis

For the synthesis of AuNTs, mixed AOT/phospholipid vesicles act as a template phase.^8^ The yield of produced gold nanoplatelets is 33% with a polydispersity of 33.3%.^8^ Because of the polydispersity, a purification step was necessary to separate the nanoplatelets from spherical particles. This was done by an increase of the AOT concentration above the critical micellization concentration (cmc) to perform a depletion flocculation leading to a sedimentation and isolation of the nanoplatelet fraction.^8^ After this purification step EDX measurements reveal (by the presence of a sulfur-peak) that AOT molecules are located on the platelet surface.^45^ Accompanying molecular dynamics (MD) simulations propose the adsorption of AOT micelles and bilayers on the {111} gold surface.^32,33^ The excess of AOT molecules in the solution could be removed by centrifugation. 79% of the nanoplatelets (including hexagonal and triangular nanoprisms) are long-term stable AOT-coated AuNTs with an average edge length of 175 ± 17 nm.^8^ A special feature of the ultrathin nanoplatelets with a thickness of 7.5 ± 1 nm, calculated by different methods, *i.e.*, X-ray reflectivity, TEM tomography,^46^ SAXS measurements,^30^ and verified in ultrafast X-ray diffraction experiments,^47^ is their long-term-stability up to a laser fluence of 2.9 mJ cm^−2^,^47^ and their ability to form monolayers.^8,31^

In the following we call these ultrathin nanoplatelets AuNTs, because of the fact that 79% of the platelets are triangularly-shaped. Note, that the term nanotriangle is often used for triangular nanoprisms, too.^48^

**Fig. 1:**
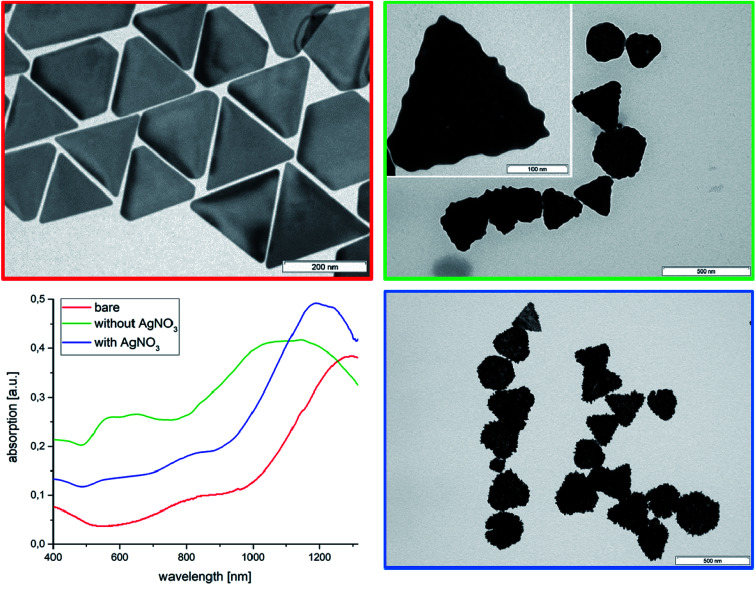
UV-vis-NIR absorption spectra with corresponding TEM micrographs of bare AuNTs (red color) in comparison to the spectra and TEM micrographs after modification in absence of AgNO_3_ (green color) and in presence of AgNO_3_ (blue color).

Due to the existence of a more rigid adsorbed AOT bilayer with a lower rate of AOT diffusion (∼10^−10^ cm^2^ s^−1^ calculated from MD simulations),^33^ which act as stabilizer, the zeta potential is −59 mV. The absorption spectrum (Fig. 1) shows a maximum at 1280 nm in the NIR-region, which is essential for *in vivo* imaging of biological tissues.^25^ A TEM micrograph of the AuNTs is shown in Fig. 1 and a low magnification SEM image in Fig. S1.†

### AuNTs surface modification in the absence of AgNO_3_.

Rough surfaces may improve the SERS enhancement factor by the lightning-rod effect and the photocatalytic activity *via* higher curvature of the surface. Therefore, we have tried to decorate the AuNTs surface with gold nanoparticles by reducing a gold chloride solution with AA. TEM micrographs of the AuNTs after treatment with the gold chloride solution in presence of AA show the formation of spherical gold nanoparticles at the surface of the triangles resulting in an undulated surface modification (Fig. 1, green marked TEM micrograph) analogous with our previous experiments by making gold half-spheres in a PEI shell of AuNTs.^49^ This process is accompanied by a shift of the UV absorption from 1320 nm of the initial bare AuNTs dispersion to the broad maximum of around 1150 nm, which can be assigned to an increase of platelet thickness. In contrast to the bare AuNTs system one can find two further peaks in the UV-vis spectrum at 580 nm and 680 nm. TEM micrographs show in addition to the undulated AuNTs significant smaller cube-like structures, which can be responsible for the UV absorption at about 600 nm. To verify this assumption, we have performed additional experiments in absence of AuNTs.

By reducing HAuCl_4_ directly with ascorbic acid in absence of AuNTs cube-like nanoparticles with small rounded spikes are formed. The UV-vis spectrum of the dispersion shows two peaks at 580 nm and at 720 nm. The first peak can be assigned to the formation of spherical particles with diameter of around 110 nm and the second one at about 720 nm can be related to star-like particles, as to be shown by the corresponding TEM micrograph (Fig. S2†).

Therefore, we can conclude that the absorption in the region between 500 and 800 nm in Fig. 1 (green curve) can be related to the formation of separately formed gold nanoparticles in solution.

### SAuNT formation in presence of AgNO_3_

Based on previous experiments,^50^ we know that in an aqueous micellar AOT solution in the presence of AgNO_3_ as a shape-directing agent and AA as reduction agent gold nanostars can be synthesized by the reduction of HAuCl_4_. Designed spikes can be synthesized in a micellar AOT solution by adding 1.5 mL of a 2 mM HAuCL_4_ and 80 μL of a 2 mM AgNO_3_ solution.^50^ In this case, it's possible to achieve particles of around 75 nm with sharpened spikes and an absorption maximum at 660 nm (compare Fig. S3†). Therefore, the following experiments were performed at similar conditions, optimized for the spike formation process.

It is well established that silver salts influence the gold nanoparticle formation. For example, the second step in the seed-mediated synthesis of nanorods is performed in presence of AgNO_3_.^51^ Recently, it was shown by Atta *et al.* that the silver nitrate concentration is of high importance for making 6-branched gold nanostars in presence of the nonionic surfactant Triton X.^10^

In presence of AgNO_3_ the AuNTs are covered by spikes, which are homogenously distributed over the whole surface area of the Au nanoplatelets (compare blue framed TEM micrograph of Fig. 1). Moreover, a small blue-shift of the absorption maximum to 1220 nm can be observed, which is related to the increased platelet thickness. The disappearance of an absorption peak maximum between 500 and 800 nm demonstrates that the gold nanoparticles are predominantly formed at the surface of the AuNTs and not in solution. This result can be understood by an electrostatic attraction between the negatively charged AOT bilayer of the triangles and the positively charged Au^3+^ and Ag^+^ ions. In consequence the metal ions are attached to the AOT bilayer surrounding the AuNTs, and the gold reduction process is performed in the AOT bilayer.

**Fig. 2:**
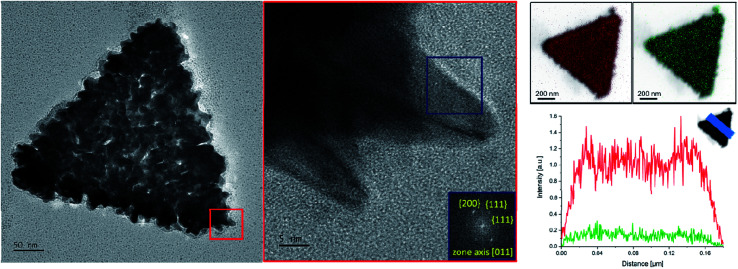
TEM micrograph of a spiked gold nanotriangle at low (scale bar: 50 nm) and high resolution (scale bar: 5 nm) with the corresponding fast Fourier transformation (FFT) of the marked area. EDX measurements of Au (red) and Ag (green) on the SAuNTs surface (right side) show fluctuations in the intensity originating from the roughness of the platelets surface due to the multitude of metal tips.

To verify the morphology of the SAuNTs, HRTEM investigations were made in combination with EDX analysis. The red area of higher magnification in Fig. 2 shows that individual spikes are formed at the platelet periphery of the nanotriangles. The associated fast Fourier transformation (FFT) in Fig. 2 (blue marked area) elucidates the crystalline structure of an individual tip. The pattern reveals a growth in [011] direction, which could also be determined for the spike growth of gold nanostars in DMF with PVP as the symmetry breaking component.^11^ The spots correspond to {200} and {111} reflections. Therefore, it can be concluded that the growth is preferred at the (100) and (111) facets leading to the formation of spikes in agreement with results obtained for nanostars.

The EDX analysis of SAuNTs (Fig. 2, right side) allows us to characterize the surface coating with the relevant metals, *i.e.*, Au (red colored) and Ag (green colored). Both metals cover the whole surface. The intensity for silver is much less pronounced and we estimate a 20% silver content in the spikes since the Au signal in Fig. 2 partially originates from the bare AuNTs without spikes, which amounts to approximately half of the volume of the SAuNTs. This is in good agreement with EDX measurements from Atta *et al.*, where the authors have found metallic silver at both side walls of the spike,^10^ as well our own EDX-line scan across a gold nanostar-spike, synthesized in concentrated aqueous AOT solution.^47^

The absence of the absorption peak around 650 ± 150 nm in the synthesis of SAuNTs in presence of AgNO_3_ is related to the fact that the whole amount of Au^3+^ ions is required for the formation of the spikes, meaning that no more precursor salt is available for making gold nanoparticles in solution.

In summary, AgNO_3_ affects the growth of the particles towards sharper spikes, which are coated by metallic silver, whereas AOT affects the growth on the AuNT surface.

For a more comprehensive discussion of the platelet stability the zeta potentials of the different systems are compared in Table 1. The samples with AgNO_3_ have a considerable higher negative zeta potential indicating a better electrostatic stabilization. The fact that silver could be found on the SAuNT surface suggests that silver builds a layer around the gold spikes. Although EDX measurements are not able to distinguish between Ag^+^ ions or Ag^0^, the increase of the negative zeta potential cannot be explained by the adsorption of positively charged Ag^+^ ions. Therefore, we assume that Ag^+^ ions are reduced on the spike surface *via* a underpotential deposition, and the corresponding NO_3_^−^ ions increase the negative zeta potential at the particle surface, which is in agreement with the results shown by Atta *et al.*^10^ and Tebbe *et al.*^6^

**Table 1:** Zeta potential measurements of the different systems with and without AuNTs

System	Zeta potential [mV]
HAuCl_4_ + AA	−15
HAuCl_4_ + AgNO_3_ + AA	−36
HAuCl_4_ + AOT + AA	−35
HAuCl_4_ + AOT + AgNO_3_ + AA	−42
AuNTs	−59
AuNTs + HAuCl_4_ + AA	−32
AuNTs + HAuCl_4_ + AgNO_3_ + AA	−39

Moreover, the use of AOT leads to a higher negative zeta potential of the AuNTs. In case of surface modification, the negative zeta potential decreases. This could be explained by the formation of gold nanoparticles in the AOT bilayer shell attached to the AuNT surface. In consequence AOT molecules are dismantled, which is accompanied by a decrease of the negative zeta potential. Nevertheless, the absolute values of the zeta potential > 30 mV are indicative of an electrostatically stabilized colloidal system. The increase of the roughness of the platelet surface leads to an additional entropic repulsion effect due to a decrease of the conformational arrangements between the particles (non-DLVO interactions).^52^

### MD simulations

Recently, we have shown by MD simulations that already after a few nanoseconds AOT bilayers adsorb on AuNT-surfaces.^33^ By adding the reducing agent AA to the system gold clusters are formed in the vicinity of the Au(111) facets, as to be shown earlier by HRTEM micrographs in the direct surrounding of gold spikes from gold nanostars.^50^

Therefore, we have performed MD simulations with a gold cluster, put into the box with water in presence of 50 AA molecules. The gold cluster is already covered with AA molecules after some MD steps, where the oxygen atoms of AA, *i.e.*, especially the 3,4-dihydroxy groups at the hydrofuran-2-on ring, come close to the gold surface (compare snapshot in Fig. S4†). This result underlines why AA is a preferred reducing component in a seed mediated synthesis of anisotropic gold nanoparticles.

**Fig. 3:**
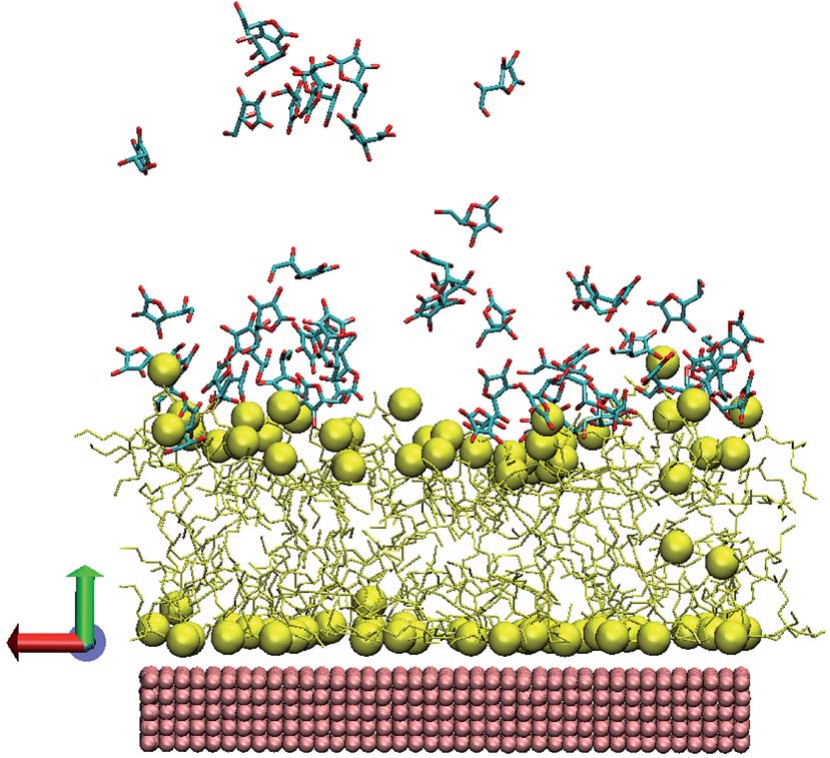
Snapshot from the last frame of MD simulation. The corresponding colors are: yellow – AOT, pink – gold surface. The AOT sulfurs and gold atoms were rendered as spheres, the water, counterions and hydrogen atoms were omitted for clarity, imitated *via* VMD package.^43^

For a more comprehensive mechanistic discussion we have now performed MD simulations by adding AA to the AOT-stabilized AuNTs. Before discussing the structural parameters, we visually inspected the layer-structure resulting in the simulations. Fig. 3 shows the snapshot extracted from the last frame after 100 ns. While the AOT layer close to the Au surface is homogeneous and flat, well-pronounced vertical deviations of AOT molecules appear in the upper layer.

To explore the roughness, the vertical deviations of sulfur atoms from the layer's center of mass were checked. Note that the parameters were calculated as described in ref. 33, which represents a vertical displacement (or average deviations) of AOT sulfur atoms from layer center of mass.

For the lower layer close to the gold we track almost no changes, while for the upper layer we see that the presence of AA molecules leads to a significant roughness of the layer. For the lower layer, the roughness value is almost the same ∼0.03 ± 0.005 nm, while for the second layer, in average, the roughness value is twice higher (∼0.23 ± 0.01 nm) than without AA (0.13 ± 0.01 nm), as compared with previous experiments.^33^ The AA molecules attached directly at the AOT headgroups of the bilayer induce large fluctuations.

**Fig. 4:**
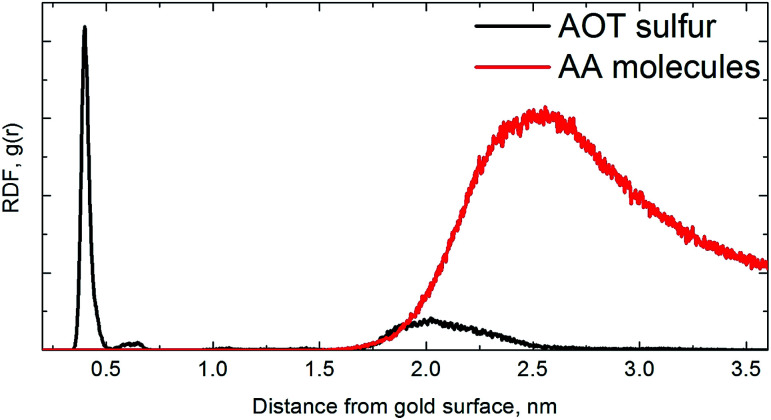
The radial distribution function (RDF) of gold surface to AA molecules and AOT sulfur atoms.

Due to the bumpy surface and orientation of AA molecules near the AOT headgroup, the bilayer thickness or calculated sulfur-to-sulfur distance is also changed from 1.76 ± 0.03 nm to 1.60 ± 0.01 nm. As a guide to interpret the numbers we plot the radial distribution function (RDF) of AA molecules (as well as AOT sulfur atoms) with respect to the gold surface in Fig. 4.

Note that the RDFs were calculated *via* the GROMACS *g_rdf* module from the last 1000 frames (last 50 ns) of both simulation and subsequently averaged over two simulations.

For AOT sulfur atoms of an AOT bilayer, it is obvious that the first layer is well-pronounced with adsorbed sulfur atoms close to gold surface as seen previously,^33^ and the second one is much more diffuse and bumpy. For AA molecules, we find one peak at the distance of ∼2.4–2.5 nm from gold surface, *i.e.*, one can conclude that the AA molecules are oriented near the fluctuating AOT head groups.

### SAuNT formation mechanism

Based on the results outlined here the mechanism of spike formation onto the AuNTs can be discussed as follows:

In a first step the positively charged Au^3+^ and Ag^+^ ions attach to the negatively charged AOT bilayer of the AuNTs by electrostatic attraction. In a second step the added AA molecules are oriented near the fluctuating AOT head groups. Therefore, the reduction process starts directly in the AOT bilayer due to preferred nucleation processes at Au(111) facets of the AuNTs.^53^ The following growth of spikes in [011] direction in the surrounding micellar AOT template phase is mainly related to the blocking of certain facets by the silver ions. Ag^+^ ions are required for the spike formation in analogy to the silver-assisted growth of gold nanorods,^54^ and the underpotential deposition of silver ions on certain gold crystal facets.^55^

### SERS performance of SAuNTs

**Fig. 5:**
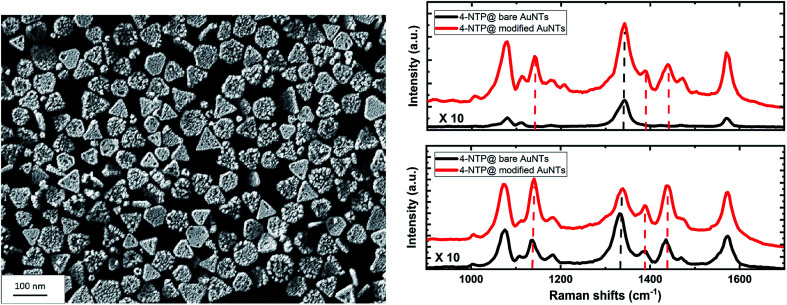
SEM micrograph of the SAuNT layer on the wafer surface (left side) with the corresponding Raman spectra (right side). Upper panel: Raman spectra measured after adsorption of 4-NTP molecules on the bare (black) and modified (red) AuNTs at a laser power of 2 mW. The intensity of the black spectrum is scaled by a factor of 10 to make it better visible. Lower panel: same for a laser power of 10 mW.

A monolayer of SAuNTs was deposited on a silicon wafer by a recently described film casting procedure.^45^Fig. 5 shows the SEM micrograph of the SAuNT-layer on the surface of the wafer. The monolayer without stacked particles is loosely packed in comparison to the “perfect” template monolayer of bare AuNTs (compare Fig. S1†), already shown by us earlier.^45^ Note that the number of regular nanotriangles is decreased from 79% to 65% during the spike formation process, which can be related to a partly deformation to non-defined structures. Therefore, the lower packing density can be related to an increased edge roughness of the SAuNTs, which leads to a repulsion between the individual nanoplatelets. Similar repulsion effects are well known from non-DLVO interactions between amphiphilic surfaces due to an entropy effect related to the surface roughness.^52^

Metal nanoparticles composed of the coinage metals (mainly gold and silver), have been used in numerous applications such as catalysis, nanomedicine, and photonics, owing to their optical properties that can be finely tuned by their localized surface plasmons.^56,57^ These collective oscillations of the particles' electron density can enhance the electric field, and therefore, the optical signals of the molecules near the particle's surfaces, *e.g. via* SERS. Moreover, the decaying surface plasmons produce highly energetic charge carriers (hot electron/hole pairs) as well as a local heat confined to the particles that are both known to initiate and/or enhance chemical transformations of the adsorbed molecules.^58,59^ These chemical transformations are termed to be plasmonically driven if the excitation wavelength lies within the plasmonic absorption band of the particles.^60^

Here we use SERS to show the plasmon-induced catalytic performance of our AuNTs. The dimerization of 4-NTP to DMAB serves as a model system of the plasmonic reaction, while the SAuNTs are compared to the AuNTs regarding the photo-catalytic reactivity and the SERS enhancement. To this end, the 4-NTP molecules were self-assembled on the nanotriangles (bare AuNTs and SAuNTs) deposited on the silicon wafers. The Raman spectrum (Fig. 5, right side) is dominated by the main three peaks of the 4-NTP at 1077, 1335, and 1575 cm^−1^, which are assigned to the C–H bending, NO_2_, and the C=C stretching modes, respectively.^60^ Fig. S5† shows the main Raman signature of the neat 4-NTP.

Fig. 5 shows the SERS spectra of 4-NTP molecules adsorbed on the surface of the AuNTs *via* the Au–S bond. The upper panel reports results measured at low laser power of 2 mW, with a very large enhancement of the Raman signature of 4-NTP molecules adsorbed on the SAuNTs (red spectrum) in comparison to those adsorbed on the bare AuNTs (black spectrum). The peak area at 1335 cm^−1^ obtained from the SAuNTs is approximately ∼20 times larger than the one obtained from the bare AuNTs, despite the loss of the signal intensity because a considerable fraction of the reactant has been transformed to DMAB molecules, which show up at 1134 cm^−1^ for the N=N stretching vibration. Consistently, the C–H bending/C–S stretching peak area at 1077 cm^−1^, which is less affected by the reaction, shows a higher enhancement by a factor of ∼75.

A direct comparison of the absolute cross-sections with the relevant literature values is difficult, because of various types of dye molecules under different reaction conditions (substrates or dispersions). For example, Scarabelli *et al.* have found an enhancement factor of 1.2 × 10^5^ by using the nonresonant benzenethiol in solution,^7^ and Kuttner *et al.* determined an EF for mercaptobenzoic acid up to 5.6 × 10^4^ at the half Au concentration.^48^ Note, that the EF in solution even compares with EF of densely packed bare NT monolayers on solid substrates.

For the bare nanotriangles, an enhancement factor of EF_AuNT_ = 2.8 × 10^4^ was found by assuming a monolayer of 4-NTP to be adsorbed on the gold surface *via* the thiol bond.^31^ It is much more difficult to estimate the surface of the SAuNTs in order to determine the enhancement factor. As a very rough estimate, we approximate the spikes as cones with an average height of 10 nm and a base diameter of 4 nm. This increases the surface by approximately 3, and yields an enhancement factor of about 8 × 10^5^ for the SAuNTs.

Next, we discuss the high plasmon-induced catalytic activity of the SAuNTs, which is evident from the appearance of strong Raman peaks at 1134, 1387, and 1434 cm^−1^ (marked with dashed lines in the red spectrum and listed in Table 2). These peaks, assigned to the C–N and N=N stretching modes of DMAB molecules, show the partial dimerization of 4-NTP into DMAB on the surface of the SAuNTs.^61^ In contrast, the reaction product is hardly visible for the bare AuNTs (black lines of Fig. 5).

**Table 2:** Raman wave numbers in relation to SERS assignments of 4-NTP and DMAB

Raman wave number (cm^−1^)	SERS assignments of 4-NTP	SERS assignments of DMAB
1077	C–H bend	
	C–S stretch	
1134		C–N stretch
1335	NO_2_ stretch	
1387, 1434		N=N stretch
1575	C=C stretch	

In order to quantify the increased reactivity of the SAuNT, we increased the laser power to 10 mW. The lower panel of Fig. 5 shows the SERS signal from SAuNT as a red spectrum. The Raman peaks of the product DMAB at 1134 and 1434 cm^−1^ even surpass the strongest peak of the reactant 4-NTP, which is the N–O stretching mode at 1335 cm^−1^. This confirms a very high reactivity of the SAuNT. We can make a relative assessment of the increased reactivity by noting that intensity ratio of the product and reactant peaks at 10 mW for the SAuNT is doubled with respect to the ratio for the AuNT. For the lower fluence of 2 mW, however, the reaction rate measured by this ratio is almost enhanced by two orders of magnitude. We tentatively attribute this large enhancement of plasmonic reactivity to the surface roughness at which the two reacting 4-NTP molecules can be arranged on the metallic surface. A quantification of the contribution of silver atoms on the surface to the reactivity and potential lightning-rod effects require additional very challenging systematic experimental investigations. Overall, we showed that our SAuNTs are a promising candidate as a plasmonic catalyst and platform for SERS.

A direct comparison between bare AuNts and SAuNTs indicates the extraordinary effect of spikes with sharp tips on SERS performance. Recently, we have shown that undulated AuNTs with gold half-spheres on the platelet surface show an intermediate enhancement effect, which is between bare AuNTs and SAuNTs. Therefore, we can conclude, that indeed the presence of spike tips on the surface of NTs is of special relevance for Raman scattering. In that case Raman active molecules, *i.e.*, 4-NTP, are sandwiched between the spike tip and a plasmonic surface, as already proposed by Rodriguez-Lorenzo *et al.*^19^

## Conclusions

In this publication, the spike formation on the surface of ultra-flat AuNTs is in the focus of research. The spike formation is controlled by the amount of AA and AgNO_3_. MD simulations show that the reducing agent, *i.e.*, AA, can directly attach to the AOT-bilayer and to gold clusters, which is of special relevance for the spike formation process in the AOT bilayer of the AuNTs, and not in solution. The final SAuNTs are covered by metallic silver on the spike surface, experimentally shown by EDX measurements. When comparing SAuNTs to bare AuNTs, we find a relative SERS signal enhancement of 75 and an increase of the plasmonic reactivity by a similar factor at low fluences, whereas at high fluences the catalytic activity is enhanced only by a factor of 2. We believe that this combination of the very intense NIR plasmon absorption band of the AuNT with an edge length of about 175 nm combined with the enhanced reactivity provided by the spikes, which are one order of magnitude smaller, are a superb platform for improving plasmonic catalysis.

## Conflicts of interest

The authors declare no competing financial interest.

## Supplementary Material

RA-010-D0RA00729C-s001
