# Supplementary material for: Spiked gold nanotriangles: formation, characterization and applications in surface-enhanced Raman spectroscopy and plasmon-enhanced catalysis
Source: RSC Adv. 2020 Feb 25;10(14):8152–60. doi: 10.1039/d0ra00729c (PMC9050016; doi:10.1039/d0ra00729c)
Supplement: RA-010-D0RA00729C-s001 [file RA-010-D0RA00729C-s001.pdf]

Electronic Supplementary Material (ESI) for RSC Advances.
This journal is © The Royal Society of Chemistry 2020

Supporting Information

Spiked gold Nanotriangles: Formation, characterization and applications in surface-enhanced Raman spectroscopy and plasmon-enhanced catalysis

Ferenc Liebig,[a] Radwan M. Sarhan,[b,c,d] Matias Bargheer,[c] Clemens N.Z. Schmitt,[e] Armen H. Poghosyan,[f] Aram A. Shahinyan,[f] Joachim Koetz[a*]

[a] Institute for Chemistry, University of Potsdam, Karl-Liebknecht-Strasse 24-25, Haus 25, 14476 Potsdam, Germany

[b] Chemistry Department, Faculty of Science, Cairo University, Cairo 12613, Egypt

[c] Institute for Physics, University of Potsdam, Karl-Liebknecht-Strasse 24-25, Haus 27, 14476 Potsdam, Germany

[d] Humboldt-Universität zu Berlin, School of Analytical Sciences Adlershof (SALSA), Albert-Einstein-Str. 5-9, 10099 Berlin, Germany

[e] Department of Biomaterials, Max Planck Institute of Colloids and Interfaces, Am Mühlenberg 1, 14476 Potsdam, Germany

[f] International Scientific –Educational Center of National Academy of Sciences, M. Baghramyan Ave. 24d, 0019 Yerevan, Armenia

Corresponding author: Joachim Koetz

E-mail: koetz@uni-potsdam.de

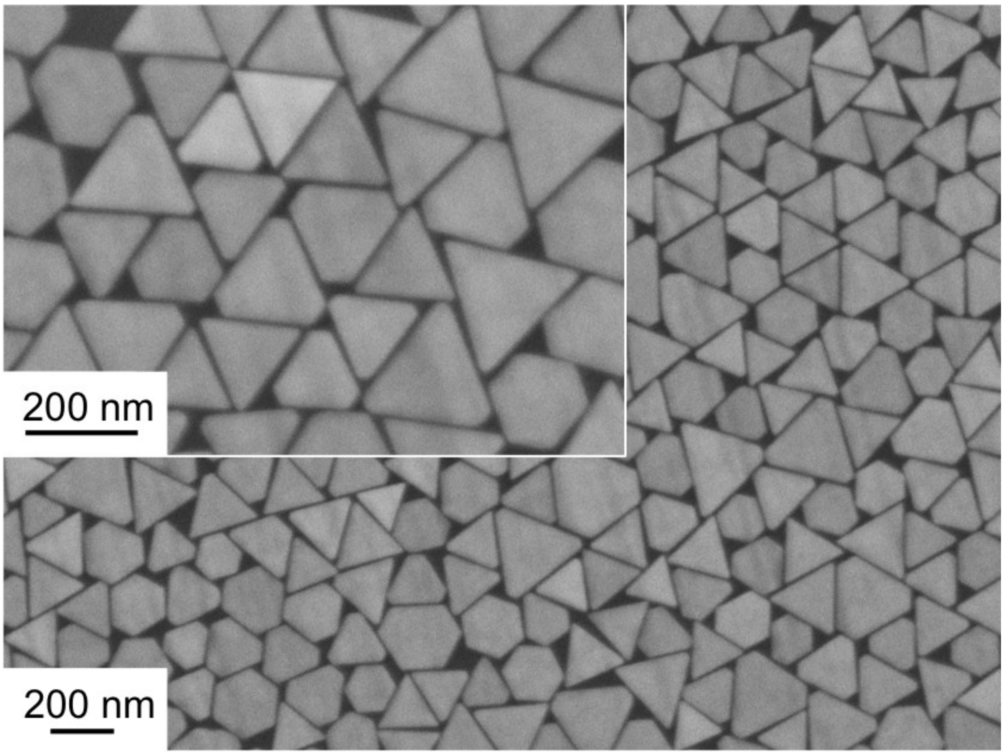

Fig. S1 Low magnification SEM micrographs of gold nanoplatelets synthesized in the AOT/phospholipid-based template phase closely packed in a “perfect” monolayer on a Si substrate.

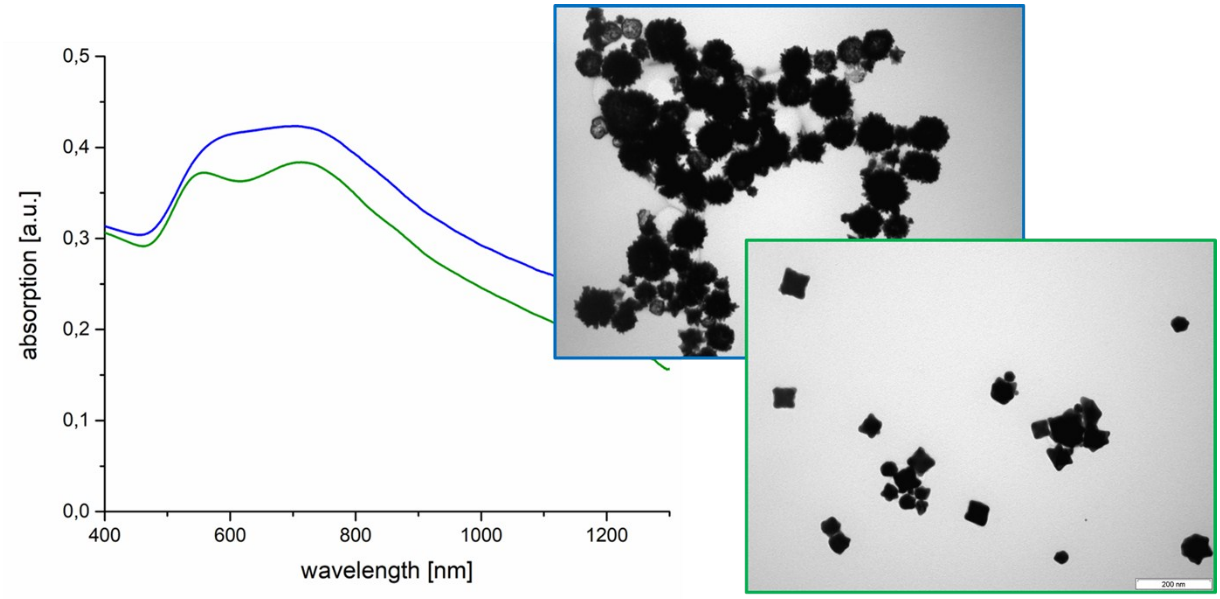

Fig. S2 TEM micrograph and the corresponding UV-vis-NIR spectrum of gold nanoparticles synthesized by the reduction of $HAuCl_4$ with AA in presence (blue) and absence (green) of $AgNO_3$.

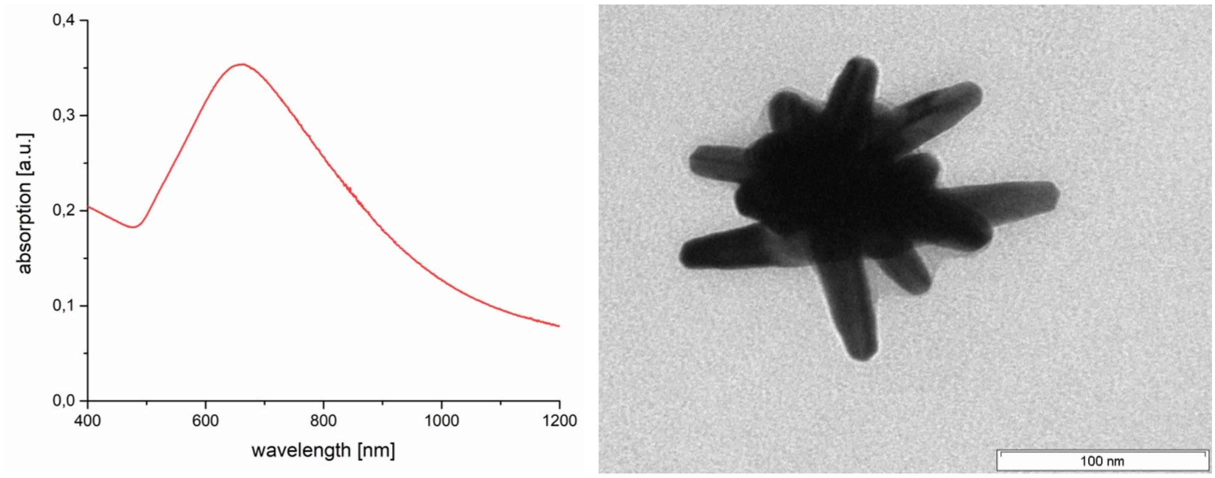

Fig. S3 TEM micrograph of a gold nanostar and the corresponding UV-vis-NIR spectrum synthesized in a concentrated AOT solution.

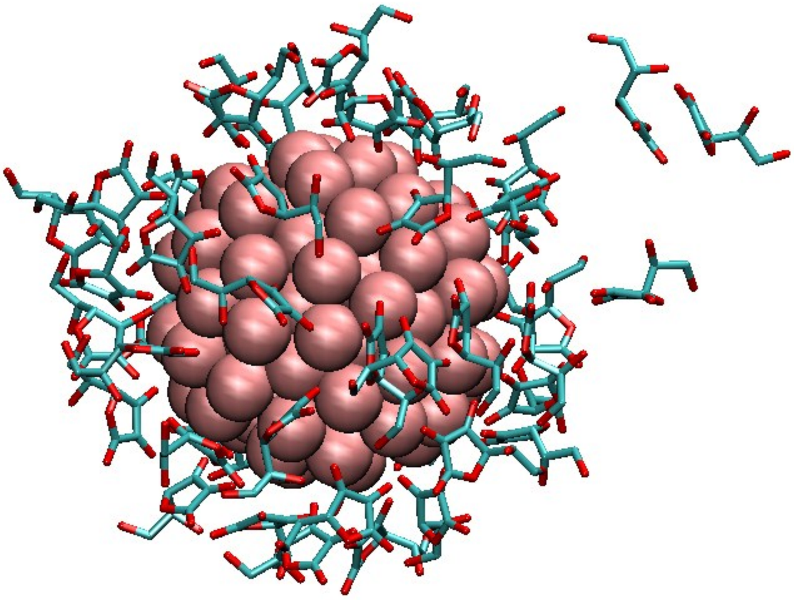

Fig. S4 Snapshot of AA molecules adsorbed at the surface of a gold cluster. The corresponding colors are: cyan – AA carbon, red – AA oxygen, pink – gold surface. The gold atoms are rendered as sphere, the water molecules and hydrogen atoms were omitted for clarity, imitated via VMD package.[36]

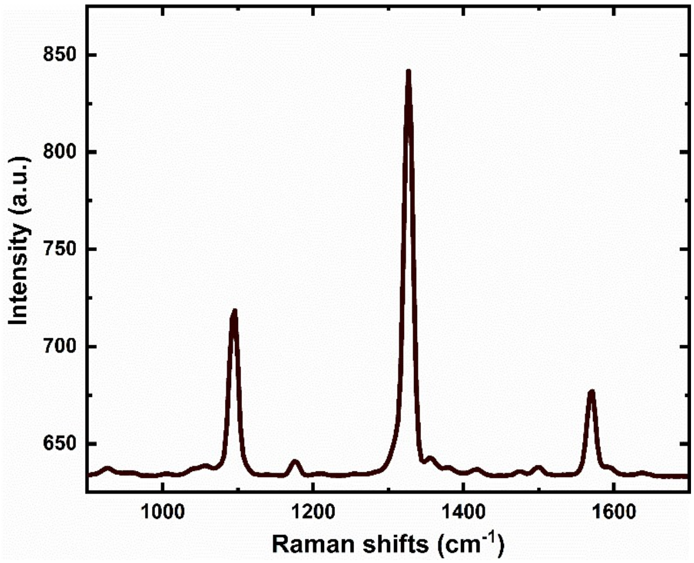

Fig. S5 Raman signatures of the neat 4-NTP molecules.
